# Visual Acuity Outcomes and Influencing Factors in a Cohort of UK Real-World Diabetic Macular Oedema Patients During the First Two Years of Anti-VEGF Treatment

**DOI:** 10.3390/pharmaceutics17010099

**Published:** 2025-01-13

**Authors:** Qing Wen, Helene Karcher, David M. Wright, Samriddhi Buxy Sinha, Usha Chakravarthy, Catarina Santos, Franklin Igwe, Recivall Salongcay, Katie Curran, Tunde Peto

**Affiliations:** 1 Centre for Public Health, Institute of Clinical Sciences, School of Medicine, Queen’s University Belfast, Belfast BT7 1NN, UK or qing.wen@lshtm.ac.uk (Q.W.); d.wright@qub.ac.uk (D.M.W.); U.Chakravarthy@qub.ac.uk (U.C.); k.curran@qub.ac.uk (K.C.); 2 Department of Non-Communicable Disease Epidemiology, Faculty of Epidemiology and Population Health, London School of Hygiene and Tropical Medicine, London WC1E 7HT, UK; 3 Novartis, Pharma AG, 4056 Basel, Switzerland; helene@alum.mit.edu (H.K.); franklin.igwe@novartis.com (F.I.)

**Keywords:** diabetic macular oedema, anti-VEGF treatment, visual acuity, injection treatment

## Abstract

Background/Objectives: The visual acuity (VA) outcomes after the first and second years of anti-vascular endothelial growth factor (anti-VEGF) treatment in patients with diabetic macular oedema (DMO) were evaluated, and the factors associated with treatment success were investigated. Methods: Using Medisoft electronic medical records (UK), this retrospective cohort study analysed VA outcomes, changes, and determinants in DMO patients at year 1 and year 2 after initial anti-VEGF injection. Descriptive analysis examined baseline demographics and clinical characteristics, while regression models were used to assess associations between these factors and changes in VA. Results: 728 DMO patients (1035 eyes) treated with anti-VEGFs (ranibizumab, aflibercept, or bevacizumab) at the Northern Ireland Mater Macular Clinic from 2008 to 2021 were evaluated. The mean age was 64.5 (SD 12.8) years, and 59.6% were male. In the first year, the median annual injection number and interval were 6.0 (IQR 5.0–8.0) and 6.1 weeks (IQR 5.4–7.8), respectively, and in the second year, they were 3.0 (IQR 2.0–5.0) and 10.0 weeks (IQR 6.5–20.1). In the first two treatment years, 83.4% and 79.8% of eyes had improved/stable VA (ISVA) respectively. The injection number, interval, baseline VA, age, and proliferative diabetic retinopathy (PDR) significantly impacted VA outcomes. Conclusions: Our study confirms the effectiveness of anti-VEGF treatments in improving or maintaining vision for DMO patients, consistent with previous real-world clinical data. An elder age, a better baseline VA, low annual injection numbers (<5), and less frequent injection intervals (≥12 weeks) were negatively associated with ISVA success in the first two years. These findings have implications for managing patient expectations, allocating resources, and understanding DMO clinical management.

## 1. Introduction

An estimated 540 million adults aged 20–79 worldwide have diabetes mellitus (DM) worldwide, and by 2045, the International Diabetes Federation (IDF) projects this number will rise to approximately 783 million, representing a 46% increase [1]. In the United Kingdom (UK), more than 5.6 million people are estimated to have DM [2,3]. Diabetic retinopathy (DR), one of the most common microvascular complications of DM, can lead to vision impairment and blindness [4,5] Diabetic macular oedema (DMO) is now the most prevalent vision-threatening form of DR, particularly among adults with type 2 DM [5]. As the population ages and the number of people with DM increases, along with a longer disease duration, DMO is becoming an increasingly critical public health issue [6,7,8,9,10].

The pathogenesis of DMO is multifactorial, involving complex pathways that contribute to the disruption of the blood–retina barrier, leading to macular oedema and/or thickening of the macula [11,12,13]. These pathological changes have been linked to hypoxia, oxidative stress, inflammation, and the subsequent upregulation of the pro-angiogenic cytokine vascular endothelial growth factor (VEGF) [11,12,13]. Elevated levels of VEGF result in impaired retinal vessel function and leakage.

Macular laser photocoagulation was historically the primary treatment for DMO before the introduction of anti-VEGF therapies. While less effective in improving visual acuity (VA) compared to anti-VEGF injections, it remains useful in stabilising vision and reducing the risk of further visual loss, particularly in non-centre-involving DMO [7,14]. Anti-VEGF injections, such as aflibercept, ranibizumab, and bevacizumab, are commonly used as the standard first-line treatment for DMO in the UK [15]. Anti-VEGF therapy serves as a highly effective approach for managing DMO, delivering rapid VA improvements, and maintaining consistent efficacy irrespective of systemic factors such as blood sugar levels, making it a reliable option even for patients with poorly controlled diabetes [16,17,18]. Randomised controlled trials (RCTs) like the RESTORE trial have demonstrated the efficacy of ranibizumab in significantly improving VA in DMO patients compared to laser photocoagulation alone [19]. Similarly, the VIVID and VISTA studies confirmed aflibercept’s superior outcomes in visual gains and retinal thickness reduction compared to laser treatment [20]. Anti-VEGF therapy requires regular intravitreal injections, typically involving an initial loading phase with monthly doses, followed by maintenance injections every 1–2 months, with intervals adjusted based on the clinical response and treatment outcomes, as recommended by the UK National Institute for Health and Care Excellence (NICE) guidelines [21,22]. This requirement places a significant burden on patients, caregivers, and healthcare systems, as it necessitates ongoing monitoring and treatment over extended periods [6,23].

The success of therapy for DMO can depend on aspects such as the severity and progression of the condition, initial vision levels, genetic traits, adherence to treatment schedules, access to care, co-existing ocular conditions, and the patient’s understanding and engagement with their treatment plan [24,25,26,27,28]. Alleviating the burden of frequent hospital visits for patients while maintaining optimised treatment outcomes has always been a goal and strategy that clinical research aims to address [23]. The aspiration to minimise the treatment demand while maximising visual outcomes has driven the evolution of anti-VEGF treatment protocols. These protocols have transitioned from fixed monthly dosing to an extension of re-treatment intervals based on repeated assessments (pro re nata) or through a modified algorithm known as “treat and extend” [20,29]. Ensuring appropriate re-treatment for maximum long-term outcomes while avoiding overtreatment is crucial for the most beneficial and sustainable strategy. Establishing new treatment paradigms requires a comprehensive understanding of visual outcomes and the factors that influence them in routine clinical care. However, data from real-world cohorts often exhibit inconsistencies, and conflicting findings have been reported [27]. Currently, there are knowledge gaps in this context, and high-quality clinical data collected during routine care are needed to provide valuable support for clinical decisions and management.

## 2. Materials and Methods

### 2.1. Study Design and Data Source

The data were from a repository of Medisoft UK electronic medical records (EMRs) captured regarding patients attending the ophthalmology clinics between 2008 to 2021 in a single tertiary referral centre in Belfast, Northern Ireland. Approval for the study was granted by the Data Guardian of the Belfast Health and Social Care Trust, UK. The EMR data consisted of patient-level longitudinal information on demographics and clinical characteristics (such as age, gender, ocular diagnoses, clinical assessment, treatments, surgeries, medications, and ophthalmic investigation details) that were recorded in a rigorously structured format. All records were fully anonymised, and dates were perturbed at the source to protect privacy.

Eligible patients were those aged 18 years or older on their first injection date (index date) who received ophthalmic care at the Mater Macula Clinic, Belfast Health and Social Care Trust (BHSCT), Northern Ireland, United Kingdom, between 1 May 2008 and 31 March 2021. The additional inclusion criteria were:

- Patients diagnosed with DMO and treated with anti-VEGF intravitreal injections (ranibizumab, aflibercept, or bevacizumab) in one or both eyes.
- Patients who completed three loading doses in the first year and at least one injection in the second year.
- Patients who had a minimum follow-up period of 24 months for the treatment of their eyes.

### 2.2. Outcomes

The primary outcomes of the study focused on VA, specifically assessing changes in VA using Early Treatment Diabetic Retinopathy Study (ETDRS) letters in the first and second years. The study also aimed to identify potential factors that could influence visual outcomes. Secondary outcomes included reporting the annual number of injections, the annual median treatment interval, the proportion of participants achieving stable or improved VA of at least one ETDRS letter (ISVA), and the proportion of participants showing an improvement of at least five ETDRS letters (IM5LR).

To evaluate the response to anti-VEGF treatment, the VA measurements at the end of each treatment year were compared with the baseline VA values. The proportion of patients with improved best-corrected visual acuity (BCVA) was also calculated. The baseline was defined as the date of the first anti-VEGF injection (index date). Baseline VA and diabetic retinopathy (DR) grades were obtained from records established on the index date or coded as missing if not available.

The VA data were recorded on different scales depending on the eye charts used during clinical assessments, including letter scores, Snellen scales, and the logarithm of the minimum angle of resolution (logMAR). The results were then exported in logMAR format. ETDRS scores were calculated using published formulas and algorithms and confirmed by standard conversion tables. Eyes with a VA measure of counting fingers [CF] or hand motion [HM] were recorded as zero ETDRS letters. For the purposes of analysis, VA improvement was defined as any increase in ETDRS letters read (≥1), worsening as a decrease in ETDRS letters (≥1), and stability as zero change in VA. VA was classified into four categories of VAs ≥ 70 ETDRS letters (the minimum driving requirement in the UK), 55–69 ETDRS letters, 35–54 ETDRS letters, and <35 ETDRS letters.

The annual number of injections and injection intervals were calculated for patients receiving anti-VEGF treatments for DMO. The injection interval, defined as the period between two adjacent injections, was not identical for each eye; therefore, median intervals were used as proxies for yearly treatment intervals in the statistical modelling. The intervals (median) were classified into three categories: less than 8 weeks (<56 days), between 8 and 11 weeks (≥56 days and <84 days), and longer than 12 weeks (≥84 days). In each of the two years, the injection number per year was stratified into three groups: 7 and greater (≥7), 5–6, and below 5 (<5), which correspond to high- (8 weeks), mid- (8–11 weeks) and low-frequency (≥12 weeks) treatment intervals, with the assumption that the injections within these groupings are distributed evenly during the observation year.

An “Intention-to-treat” approach was adopted to handle missing values for VA and DR grades. Missing values were imputed using the last-observation-carried-forward (LOCF) or next-observation-carried backward (NOCB) method to determine the closest measurement as a substitute. If no records were available for LOCF or NOCB, multiple imputations were carried out. Missing data were imputed using chained equations with 500 iterations, creating 20 imputed datasets. The final results were calculated using Rubin’s rules, accounting for the variability within the imputed datasets.

### 2.3. Statistical Analysis

Descriptive analyses were conducted for demographic and clinical characteristics. Baseline demographic and clinical characteristics, including age, gender, DM type, and DMO laterality (unilateral/bilateral), were summarised at the patient level. The eye was the unit of analysis for baseline VA and DR/DMO grades, the injection number, and injection intervals.

Baseline characteristics are presented as a mean (standard deviation [SD]) or median (interquartile range [IQR]) for continuous variables and as frequency (percentage) for categorical variables. The number of injections, injection intervals, and changes in VA between the baseline and last injection for the first and the second year of treatment were calculated. Demographic characteristics of eyes in groups attaining different levels of annual injection numbers and median intervals were compared by conducting a one-way ANOVA for age and Pearson’s chi-squared tests (χ2) for other variables. Histograms, normal quantile plots (QQ plots), and the Shapiro normality test were used to test data normality.

Simple and multiple analyses were conducted using Generalised Linear Models (GLM) with a logit link function to investigate the effects of covariates (baseline demographic and clinical factors, such as age and the number of anti-VEGF injections) on VA response at 1-year and 2-year intervals. The associations between baseline demographic and clinical factors, and VA outcomes for each eye were examined, with adjustments made for other covariates. Generalised estimating equation (GEE) methods with an exchangeable correlation structure were applied to account for inter-eye correlation within the same patient and to identify the determinants of achieving ISVA. The potential factors that were investigated included age, gender, DM type, baseline VA, baseline DR grades, and median injection interval during the two years of follow-up. All seven variables were included in the multiple models; odds ratios and 95% CIs were reported. Box–Tidwell tests, graphical methods, and Variance Inflation Factor (VIF) tests were used to assess regression assumptions and multicollinearity in the data.

Sensitivity analyses based on the GLM models were performed to evaluate the robustness of the main findings. These included complete case analysis; treating missing values as a separate category; models excluding either GEE and/or multiple imputation; and using the total number of injections instead of the median injection interval as a proxy for injection frequency. In addition to analysing ISVA outcomes, we examined whether patients achieved an increase in VA of at least five EDTRS letters during the first and second years, providing further insight into outcomes under a more rigorous criterion for VA improvement.

All statistical analyses were performed in R (version 3.6.1, R Foundation for Statistical Computing, Vienna, Austria). The platform was x86_64-w64-mingw32/x64 (64-bit). The R library mitml 0.4.3 and geepack 1.3.3 packages were used for the multiple imputation routines and GEE modelling.

## 3. Results

### 3.1. Study Population

A total of 728 Type 1 or Type 2 DM patients (1035 eyes) with a diagnosis of DMO who fulfilled all eligibility criteria were identified in the EMR and included in the study. Among them, 307 (42.2%) patients had both eyes affected by DMO, while 421 (57.8%) had only one eye affected. At the initial encounter, the patients had a mean age of 64.5 (SD 12.8) years, ranging from 23 to 93 years old. More patients were males (n = 434, 59.6%), and more than three-quarters had Type 2 DM (n = 587, 80.6%). The cohort had a similar distribution of right- (49.2%) and left-eyes (50.8%). The mean baseline VA was 61.5 (SD 16.3) ETDRS letters. Approximately half of the eyes had a good VA of 70 or more ETDRS letters, and less than 6.5% had a poor VA of less than 35 ETDRS letters, indicating severe vision loss. Mild DR was present in 21.2% of the eyes, while 41.5% had moderate to severe DR and 19.6% had PDR. The severity of DR was not available for under one-fifth (17.7%) of the eyes (Table 1).

### 3.2. Anti-VEGF Agents, Injection Numbers, and Injection Intervals

The anti-VEGF agents used in the cohort were ranibizumab (Lucentis), aflibercept (Eylea), or off-label bevacizumab (Avastin), though this latter drug was used infrequently. Of the 1035 eyes, 761 (73.5%) received exclusive monotherapy with either ranibizumab (58.2%) or aflibercept (41.8%). The remaining 274 (26.5%) eyes had switched treatment at least once within the first two years of anti-VEGF therapy.

A total of 6399 and 3798 anti-VEGF injections were administered to the entire cohort in the first and second treatment years, respectively. The median number of injections was 6.0 (IQR 5.0–8.0) in the first year and 3.0 (IQR 2.0–5.0) in the second year. In the first year, nearly 80% of eyes (n = 789) received five or more injections, while this proportion dropped to 32.4% (n = 335) in the second year. Only a few eyes (n = 71 and n = 18 in the first and second years, respectively) received a high-frequency anti-VEGF injection (10 or more injections over 1 year), approaching monthly dosing (Figure S1).

When the cohort was grouped by injection re-treatment intervals (median) of <8 weeks, 8 to 11 weeks, and ≥12 weeks, which represented high-, mid-, and low-frequency groups, it was found that higher proportions received more frequent treatment in the first year than in the second year. The treatment intervals in the first year and the second year were <8 weeks in 791 (76.4%) eyes and 383 (37.0%) eyes, 8–11 weeks in 181 (17.5%) eyes and 194 (18.7%) eyes, and 12+ weeks in 63 (6.1%) eyes and 458 (44.3%) eyes, respectively. The overall median injection interval was 6.1 weeks in year 1 versus 10.0 in year 2. The injection numbers by group in each year exhibit patterns that are consistent with the interval grouping. There were no significant associations between the total number of injections in the first or second year and factors including gender (χ2 test, ^p^_Y1_ = 0.098, ^p^_Y2_ = 0.65), DM types (χ2 test, ^p^_Y1_ = 0.71, ^p^_Y2_ = 0.71), DR grades (χ2 test, ^p^_Y1_ = 0.81, ^p^_Y2_ = 0.21), and age (ANOVA, ^p^_Y1_ = 0.26, ^p^_Y2_ = 0.48). However, the baseline VA showed a marginal correlation (χ2 test, ^p^_Y1_ = 0.05, ^p^_Y2_ = 0.08). These findings apply to injection intervals in the first and second years. Only baseline VA had a marginally significant effect, as shown by a χ2 test (χ2 test, ^p^_Y1_= 0.01, ^p^_Y2_ = 0.045). In contrast, gender (χ2 test, ^p^_Y1_ = 0.43, ^p^_Y2_ = 0.25), DM types (χ2 test, ^p^_Y1_ = 0.46, ^p^_Y2_ = 0.11), DR grades (χ2 test, ^p^_Y1_ = 0.22, ^p^_Y2_ = 0.99), and age (ANOVA, ^p^_Y1_ = 0.58, ^p^_Y2_ = 0.94) did not demonstrate a significant association with injection intervals.

### 3.3. Visual Acuity Outcomes

**Figure 1:**
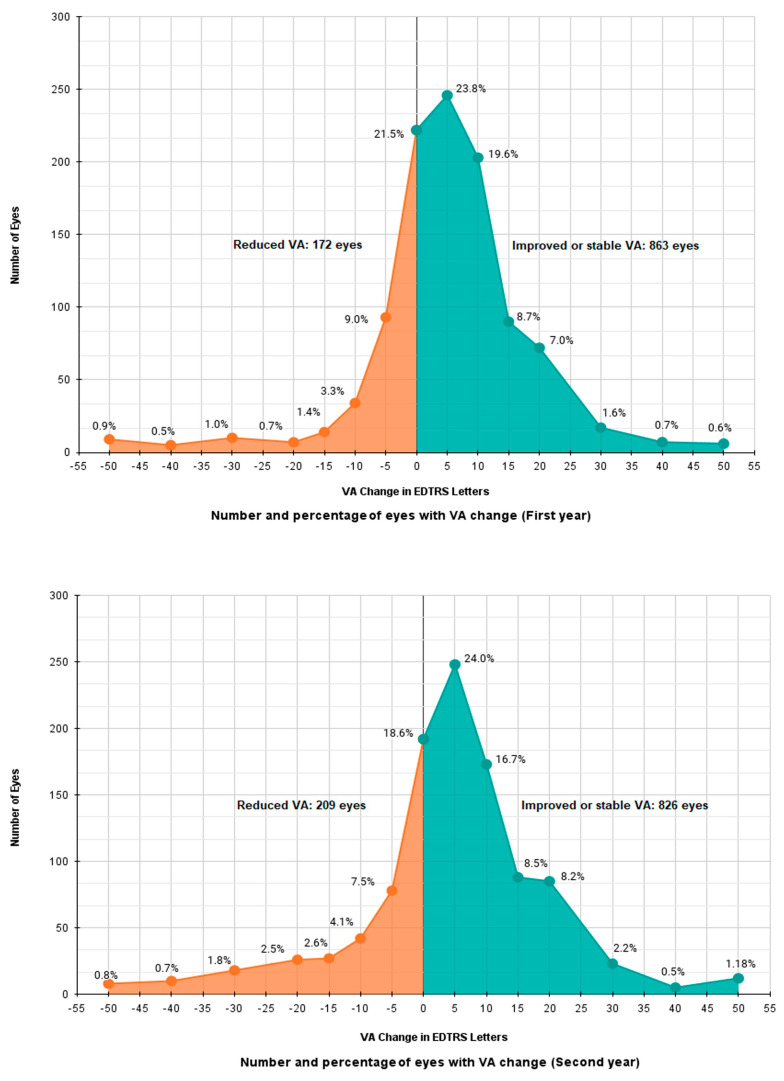
Number and percentage of eyes with VA change in the first and second year.

In the first year, more than half of the eyes (n = 613, 59.2%) achieved a good VA (≥70 letters). The mean improvement in VA (SD) was +5.1 (12.2) ETDRS letters from a baseline of 61.5 ETDRS letters. Similarly, in the second year, 597 (57.8%) eyes achieved ≥70 letters. The mean (SD) VA gain was +4.5 (14.3) ETDRS letters from the baseline, which was slightly lower than in the first year. After the first year of treatment, a considerable number of eyes (n = 863, 83.4%) showed improved or stable VA. This number moderately decreased in the second year (n = 826, 79.8%). Figure 1 displays the distribution of VA changes in the first and second years of treatment. The frequency plot peaks at around five letters for both years, with a slight increase in the number of eyes experiencing greater than a one-letter loss in year 2. The percentage of eyes showing improvement remained relatively stable across the years, whereas the proportion of eyes experiencing worsening increased notably in the second year. During the first year of treatments, over 60% of eyes (n = 641) achieved an improvement of ≥5 ETDRS letters. Among them, 38.2% (n = 395) improved by ≥10 ETDRS letters, and 18.6% (n = 182) eyes showed an improvement of at least 15 ETDRS letters. Around one-fifth of eyes (n = 172, 16.6%) experienced a loss of ≥5 ETDRS letters, and among them, 79 (7.6%) lost ≥10 ETDRS letters. Likewise, in the second year, ≥5 ETDRS letters of VA improvement were observed in more than 60% of eyes (n = 634), of which 386 (37.3%) eyes gained ≥10 ETDRS letters and 213 (20.6%) gained ≥15 letters. Additionally, 209 (20.2%) eyes experienced a decline of ≥5 ETDRS letters, while 131 (12.7%) eyes experienced a decline of ≥10 ETDRS letters.

**Figure 2:**
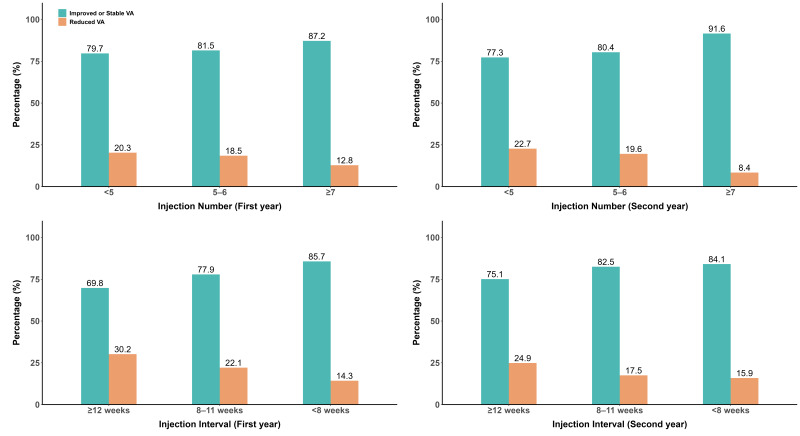
Percentages of VA changes by injection numbers/intervals in the first and second year.

The group of eyes with the highest frequency of injections, falling into the shortest treatment interval category, had the highest percentage of improvement in ISVA, with 85.7% achieving good VA gains, compared to 69.8% of eyes in the longest interval group. Among those who continued with an injection interval of <8 weeks, the highest proportion of VA gainers was observed in the second year, with 84.1% achieving good VA gains, followed by those with an 8–11-week interval (82.5%) and, lastly, by those in the ≥12 weeks group (75.1%) (Figure 2). It is also the case that when eyes were grouped according to the annual number of injections received (≥7, 5–6, and below 5), the proportion achieving ISVA exhibited a declining trend from the high- to low-injection-number group. Table 2 demonstrates the distribution of ISVA achievements for the categories of baseline VA, DR grades, injection intervals, and numbers.

### 3.4. Factors Associated with Visual Acuity Change

The factors included age, gender, DM type, injection interval, injection numbers, baseline VA, and DR grades, whose associations with VA outcomes were assessed using marginal models fitted with GEE to account for inter-eye correlation. After adjusting for other covariates, the injection interval, injection numbers, baseline VA, and age were found to be significantly associated with the outcomes of ISVA at the end of both the first and second treatment years. No violations were detected in the tests for regression assumptions.

For each one-year increase in baseline age, the odds of improvement in ISVA decreased by about 2.0% in both years (OR: 0.98, 95% CI: 0.97–0.99, ^p^_Y1_ = 0.042, ^p^_Y2_ = 0.026). The overall DR grade did not significantly impact ISVA in the first year (^p^_Y1_ = 0.44) but had marginal significance in the second year (^p^_Y2_ = 0.07). Additionally, in the second year, the odds of attaining ISVA outcomes among eyes with PDR decreased by 45.6% (^p^_Y2_ = 0.02, OR: 0.54, 95% CI: 0.31–0.95) compared to eyes with background retinopathy (mild–moderate).

**Figure 3:**
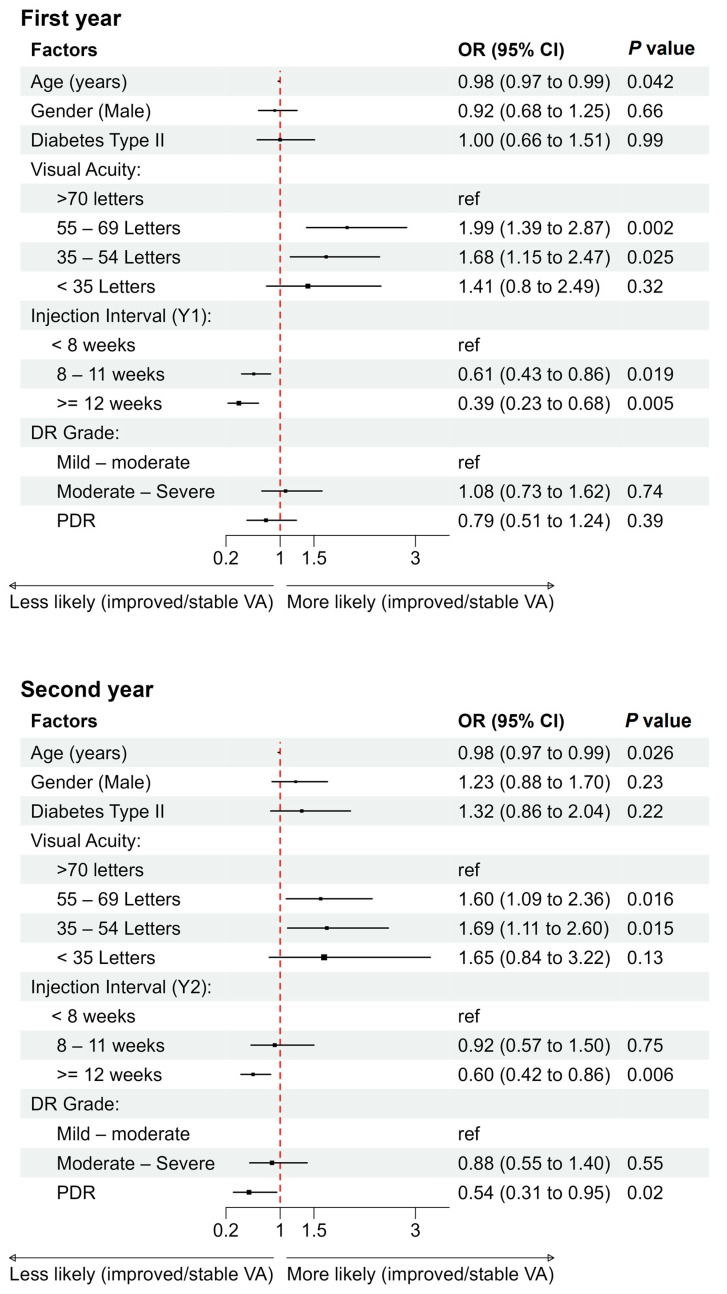
Forest Plots of associations between explanatory factors and odds of improved/stable VA in the first and second year.

After controlling for other covariates, it was found that eyes with a baseline VA between 55 and 69 ETDRS letters had the highest probability of achieving ISVA (^p^_Y1_ = 0.002, OR: 1.99, 95% CI: 1.39–2.86) in the first year, compared to those with a baseline VA of ≥70 ETDRS letters. Conversely, eyes with a baseline VA of 35 to 54 letters had a reduced probability of achieving first-year ISVA (^p^_Y1_ = 0.025, OR: 1.68, 95% CI: 1.15–2.47). For second-year ISVA, eyes with a baseline VA of 55–69 ETDRS letters (^p^_Y2_ = 0.016, OR: 1.60, 95% CI: 1.09–2.36) had slightly lower odds of achieving improvement than those with a baseline VA of 35–54 ETDRS letters (^p^_Y2_ = 0.015, OR:1.69, 95% CI: 1.11–2.60). No significant differences were found for those with a baseline VA < 35 letters compared to those with a VA ≥ 70 ETDRS letters (^p^_Y1_ = 0.32, ^p^_Y2_ = 0.13) in both years (Figure 3).

The likelihood of achieving ISVA was higher for the shortest treatment interval. In comparison to an interval of less than 8 weeks, the odds of achieving ISVA for the longest injection interval (interval ≥ 12 weeks) decreased by 61.0% (^p^_Y1_ = 0.005, OR: 0.39, 95% CI: 0.23–0.68) and 40.1% (^p^_Y2_ = 0.006, OR: 0.60, 95% CI: 0.42–0.86) in the first and second year, correspondingly. For the interval of 8–11 weeks, the odds of success decreased by 39.0% (^p^_Y1_ = 0.019, OR: 0.61, 95% CI: 0.43–0.86); in the first year, however, there were no significant differences in the second year (^p^_Y2_ = 0.75).

The use of injection numbers as a predictor in the model yielded similar results as the use of intervals. In both simple and multiple models, the injection numbers were positively correlated with ISVA outcomes. Compared to those with the most frequent level of injections (≥7), both categories of eyes with fewer or the fewest injections (5–6 or <5 injections) were likely to have worse VA outcomes in the first year (^p^_Y1_ = 0.048, OR: 0.67, 95% CI: 0.48–0.93; ^p^_Y1_ = 0.032, OR: 0.62, 95% CI: 0.43–0.90) and second year (^p^_Y2_ = 0.019, OR: 0.44, 95% CI: 0.25–0.78; ^p^_Y2_ = 0.001, OR: 0.35, 95% CI: 0.21–0.59) (Table S1).

The factors influencing the achievement of a higher threshold of VA improvement, defined as an improvement of at least five ETDRS letters (IM5LR), were similar to those observed during the initial stable visual acuity (ISVA) assessments (Table S2). The injection numbers, injection interval, age, and baseline VA were found to be significantly correlated with the outcome of achieving IM5LR. In both the first and second year, eyes with the lowest baseline VA (<35 ETDRS letters) had the highest odds of achieving IM5LR, whereas eyes with the highest baseline VA (≥70 ETDRS letters) showed a decline in achievement. Compared to eyes with a high treatment frequency (interval < 8 weeks), eyes with a low treatment frequency (interval ≥ 12 weeks) had decreased odds of achieving IM5LR in both the first and second years (first year: ^p^_Y1_ = 0.006, OR: 0.40, 95% CI: 0.23–0.69; second year: ^p^_Y2_ < 0.001, OR: 0.58, 95% CI: 0.45–0.74). Similarly, the low injection group (<5) had lower odds of achieving IM5LR compared to the high injection group (≥7). This was also observed for the injection intervals of 8–11 weeks (^p^_Y1_ = 0.014) and injection numbers of 5–6 (^p^_Y1_ = 0.004) in the first year, which were negatively correlated with the IM5LR outcomes when compared to high-frequency or high-number injections, respectively. In summary, eyes with a low injection frequency (interval of 12 weeks or more) had decreased odds of achieving both ISVA and IM5LR compared to eyes with a high frequency (interval of less than 8 weeks) in both the first and second years. A decrease in odds of achieving ISVA and IM5LR was also observed for eyes receiving less than five injections annually, compared to eyes receiving seven or more injections. The findings from the sensitivity analyses aligned with the main results.

## 4. Discussion

This study provides real-world evidence on the effectiveness of anti-VEGF therapy for DMO patients, revealing that visual gains achieved in the first year are sustained into the second year. While approximately 80% of eyes maintained or improved VA, our results align with previous research showing that real-world outcomes tend to fall short of those seen in clinical trials, likely due to the less frequent injections in routine practice. This highlights the ongoing challenge of achieving optimal treatment outcomes in real-world settings [29,30,31,32].

Identifying prognostic factors is important in assisting clinicians in making decisions about whether to extend the frequency of treatment after initiation, as not all DMO patients respond equally well to intravitreal anti-VEGF therapy. Our data show that the injection number, injection frequency, baseline VA, and age are important factors that contribute to improving and maintaining visual outcomes, while the impact of gender and DM type is less significant.

Younger patients and those with a lower baseline VA were more likely to experience greater VA improvements, consistent with prior studies [27]. Additionally, our data highlight the critical role of injection frequency: patients receiving fewer than five injections annually or with intervals of 12 weeks or more between injections were more likely to experience poorer visual outcomes. These findings suggest that treatment regimens with shorter intervals and more frequent injections could improve VA outcomes in DMO patients.

The study also underscores the potential benefits of longer-acting anti-VEGF treatments, particularly for patients who struggle with frequent clinic visits due to comorbidities or logistical challenges. To address this treatment burden, emerging options, such as longer-acting anti-VEGF formulations and sustained-release steroid implants, are being investigated [33]. Combining pharmacological treatments with laser photocoagulation (either focal or macular grid laser) also presents a potential therapeutic strategy [34]. New therapies aimed at enhancing the durability and efficacy of anti-VEGF treatments are showing promise in reducing the frequency of retreatments. For instance, the KESTREL and KITE phase 3 trials assessed brolucizumab in patients with DMO [35]. At 100 weeks, brolucizumab showed comparable VA gains in comparison to aflibercept, but with fewer injections, as nearly 50% of KITE patients were able to extend treatment intervals to 12 or 16 weeks [35]. Both trials demonstrated favourable safety outcomes, with no new cases of retinal vasculitis, although intraocular inflammation rates were slightly higher for brolucizumab. Similarly, the YOSEMITE and RHINE trials on faricimab showed it was as effective as aflibercept, with 50% of patients achieving 16-week dosing intervals and a well-tolerated safety profile [36]. As newer therapeutic options are developed, ensuring that patients can maintain consistent care without compromising efficacy will be essential.

### 4.1. Strengths

The key strength of this study lies in its examination of treatment patterns and their effects in real-life situations. This focus on real-world data enhances the relevance and applicability of the findings, providing deeper insights into treatment efficacy beyond the confines of controlled clinical trials. We used data from a well-designed EMR system, and the resulting data are representative of the treatment protocols used in routine care. Our study is longitudinal and based on detailed treatment records for individual patients, who were treated at a single clinical facility with similar management routines based on agreed-upon protocols. VA measurements were made using harmonised methods and standardised equipment. In our analysis, we used both injection numbers and intervals (median), which complementarily cover distinctive aspects of treatment patterns. We also classified treated eyes using injection interval categories (<8 weeks, between 8 and 11 weeks, ≥12 weeks) to facilitate the interpretation of findings, which can be easily applied in clinical decision-making.

### 4.2. Limitations

Due to the inherent nature of the EMR data collected during routine care, our study has several limitations. Our data are derived from a single clinic, which may limit the generalisability to other settings. Moreover, the retrospective design, the absence of information on the type or location of DMO, and clinical variables like HbA1c, PDR severity, a lack of differentiation between anti-VEGF agents and their dosing regimens, and a reliance on clinical judgment for excluding tractional DMO may introduce potential sources of bias. Despite these limitations, the use of a well-structured EMR system and a large sample size strengthens the validity of our findings.

## 5. Conclusions

In conclusion, this study reinforces the value of anti-VEGF therapy in maintaining visual function in people with DMO. The identified factors, such as injection frequency and baseline VA, provide important guidance for clinical decision-making. Further research is needed to explore longer-acting and more effective treatments for optimising injection intervals, with the potential to improve both patient outcomes and quality of life.

## Figures and Tables

**Table 1:** Baseline demographic and clinical characteristics of DMO patients and their injections in the 1st and 2nd year.

**Baseline Demographic**	Patients (n = 728)	
Gender, n (%): Male	434 (59.6)	
Age: Mean (SD)	64.5 (12.8)	
Laterality, n (%): Unilateral	421 (57.8)	
Diabetes Type, n (%): Type II	587 (80.6)	
**Baseline Clinical Characteristics**	Eyes (n = 1035)	
Eyes: Right, n (%)	509 (49.2)	
Baseline visual acuity: ETDRS mean (SD)	61.5 (SD: 16.3)	
≥70 letters, n (%)	463 (44.7)	
55–69 letters	306 (29.6)	
35–54 letters	199 (19.2)	
<35 letters	67 (6.47)	
Baseline DR grades: Mild–moderate, n (%)	219 (21.2)	
Moderate to Severe	430 (41.5)	
PDR	203 (19.6)	
Missing	183 (17.7)	
**Anti-VEGF Injections**	1st year	2nd year
Injection interval (wks), median, IQR	6.1 (5.4–7.8)	10.0 (6.5–20.1)
<8 weeks, n (%),	791 (76.4)	383 (37.0)
8–11 weeks, n (%),	181 (17.5)	194 (18.7)
≥12 weeks, n (%),	63 (6.1)	458 (44.3)
Injection numbers, median, IQR	6.0 (5.0–8.0)	3.0 (2.0–5.0)
≥7, n (%)	422 (40.8)	142 (13.7)
5–6, n (%)	367 (35.5)	193 (18.7)
<5, n (%)	246 (23.8)	700 (67.6)

**Table 2:** Clinical characteristics of DMO eyes and VA changes in the first and second year.

Clinical Characteristics|VA Changes	1st Year		2nd Year	
	Improved/Stable	Reduced	Improved/Stable	Reduced
Eyes (Total = 1035), n (%)	863 (83.4)	172 (16.6)	826 (79.8)	209 (20.2)
**Baseline Visual Acuity:** ETDRS letters, n (%)				
≥70 letters	367 (79.3)	96 (20.7)	353 (76.2)	110 (23.8)
55–69 letters	269 (87.9)	37 (12.1)	253 (82.7)	53 (17.3)
35–54 letters	172 (86.4)	27 (13.6)	166 (83.4)	33 (16.6)
<35 letters	55 (82.1)	12 (17.9)	54 (80.6)	13 (19.4)
**DR Grades,** n (%)				
Mild–moderate	183 (21.2)	36 (20.9)	184 (84.0)	35 (16.0)
Moderate to severe	364 (42.2)	66 (38.4)	355 (82.6)	75 (17.4)
PDR	167 (19.4)	36 (20.9)	154 (75.9)	49 (24.1)
Missing	149 (17.3)	34 (19.8)	133 (72.7)	50 (27.3)
**Injection Interval**, n (%)				
<8 weeks	678 (85.7)	113 (14.3)	322 (84.1)	61 (15.9)
8–11 weeks	141 (77.9)	40 (22.1)	160 (82.5)	34 (17.5)
≥12 weeks	44 (69.8)	19 (30.2)	344 (75.1)	114 (24.9)
**Injection Numbers**, n (%)				
≥7	368 (87.2)	54 (12.8)	130 (91.6)	12 (8.4)
5–6	299 (81.5)	68 (18.5)	155 (80.4)	38 (19.6)
<5	196 (79.7)	50 (20.3)	541 (77.3)	159 (22.7)

n = Number of eyes achieving VA outcomes in each category; % = (n/total for each category) × 100.

## Data Availability

The original contributions presented in this study are included in the article/Supplementary Material. Further inquiries can be directed to the corresponding author.

## Supplementary Materials

The following supporting information can be downloaded at: https://www.mdpi.com/article/10.3390/pharmaceutics17010099/s1, Figure S1: The distribution of the number of injections in the first and second year; Table S1: Marginal Models with GEE for Analysing ISVA and Predictors including injection numbers in the 1st and 2nd Year (n = 1035); Table S2: Marginal models with GEE to analyse injection interval/number and other potential predictors associated with IM5LR in the 1st and 2nd Year (n = 1035) (Full model).

## References

[1] IDF Atlas 10th Edition IDF Diabetes Atlas. https://diabetesatlas.org/idfawp/resource-files/2021/07/IDF_Atlas_10th_Edition_2021.pdf.

[2] Zondervan B.Y.M., Bascaran C., Sandi F., Cornes C., Williams P. (2018). Diabetes and diabetic retinopathy: Changes in understanding of the disease over the last 25 years and how the UK is helping. Eye News.

[3] Diabetes UK How Many People in the UK Have Diabetes? 2023. https://www.diabetes.org.uk/about-us/about-the-charity/our-strategy/statistics.

[4] Cheung N., Mitchell P., Wong T.Y. (2010). Diabetic retinopathy. Lancet.

[5] Tan G.S., Cheung N., Simó R., Cheung G.C.M., Wong T.Y. (2017). Diabetic macular oedema. Lancet Diabetes Endocrinol..

[6] Cheung N., Cheung C.M.G., Talks S.J., Wong T.Y. (2020). Management of diabetic macular oedema: New insights and global implications of DRCR protocol V. Eye.

[7] Stefanickova J., Cunha-Vaz J., Ulbig M., Pearce I., Fernández-Vega Sanz A., Theodossiadis P., Kodjikian L., Izmailov A., Muston D., Vassilev Z. (2018). A noninterventional study to monitor patients with diabetic macular oedema starting treatment with ranibizumab (POLARIS). Acta Ophthalmol..

[8] Vision Loss Expert Group of the Global Burden of Disease Study, The GBD 2019 Blindness and Vision Impairment Collaborators (2024). Global estimates on the number of people blind or visually impaired by diabetic retinopathy: A meta-analysis from 2000 to 2020. Eye.

[9] Bourne R.R.A., Jonas J.B., Bron A.M., Cicinelli M.V., Das A., Flaxman S.R., Friedman D.S., Keeffe J.E., Kempen J.H., Leasher J. (2018). Prevalence and causes of vision loss in high-income countries and in Eastern and Central Europe in 2015: Magnitude, temporal trends and projections. Br. J. Ophthalmol..

[10] Teo Z.L., Tham Y.C., Yu M., Chee M.L., Rim T.H., Cheung N., Bikbov M.M., Wang Y.X., Tang Y., Lu Y. (2021). Global Prevalence of Diabetic Retinopathy and Projection of Burden through 2045: Systematic Review and Meta-analysis. Ophthalmology.

[11] Ciulla T.A., Amador A.G., Zinman B. (2003). Diabetic retinopathy and diabetic macular edema: Pathophysiology, screening, and novel therapies. Diabetes Care.

[12] Bahrami B., Zhu M., Hong T., Chang A. (2016). Diabetic macular oedema: Pathophysiology, management challenges and treatment resistance. Diabetologia.

[13] Ehrlich R., Harris A., Ciulla T.A., Kheradiya N., Winston D.M., Wirostko B. (2010). Diabetic macular oedema: Physical, physiological and molecular factors contribute to this pathological process. Acta Ophthalmol..

[14] Mueller I., Talks J.S. (2022). Should we still be performing macular laser for non-centre involving diabetic macular oedema? No. Eye.

[15] Lanzetta P., Loewenstein A., Vision Academy Steering Committee (2017). Fundamental principles of an anti-VEGF treatment regimen: Optimal application of intravitreal anti-vascular endothelial growth factor therapy of macular diseases. Graefes. Arch. Clin. Exp. Ophthalmol..

[16] Ollendorf D.A., Colby J.A., Pearson S.D. (2013). Comparative effectiveness of anti-VEGF agents for diabetic macular edema. Int. J. Technol. Assess. Health Care.

[17] Li Y.F., Ren Q., Sun C.H., Li L., Lian H.D., Sun R.X., Su X., Yu H. (2022). Efficacy and mechanism of anti-vascular endothelial growth factor drugs for diabetic macular edema patients. World J. Diabetes.

[18] Bansal A.S., Khurana R.N., Wieland M.R., Wang P.W., Van Everen S.A., Tuomi L. (2015). Influence of Glycosylated Hemoglobin on the Efficacy of Ranibizumab for Diabetic Macular Edema: A Post Hoc Analysis of the RIDE/RISE Trials. Ophthalmology.

[19] Mitchell P., Bandello F., Schmidt-Erfurth U., Lang G.E., Massin P., Schlingemann R.O., Sutter F., Simader C., Burian G., Gerstner O. (2011). The RESTORE study: Ranibizumab monotherapy or combined with laser versus laser monotherapy for diabetic macular edema. Ophthalmology.

[20] Brown D.M., Schmidt-Erfurth U., Do D.V., Holz F.G., Boyer D.S., Midena E., Heier J.S., Terasaki H., Kaiser P.K., Marcus D.M. (2015). Intravitreal aflibercept for diabetic macular edema: 100-week results from the VISTA and VIVID studies. Ophthalmology.

[21] NICE Aflibercept for Treating Diabetic Macular Oedema. https://www.nice.org.uk/guidance/ta346.

[22] NICE Ranibizumab for Treating Diabetic Macular Oedema. https://www.nice.org.uk/guidance/ta274.

[23] Sivaprasad S., Oyetunde S. (2016). Impact of injection therapy on retinal patients with diabetic macular edema or retinal vein occlusion. Clin. Ophthalmol..

[24] Liu E., Craig J.E., Burdon K. (2017). Diabetic macular oedema: Clinical risk factors and emerging genetic influences. Clin. Exp. Optom..

[25] Cheema A.A., Cheema H.R. (2024). Diabetic Macular Edema Management: A Review of Anti-Vascular Endothelial Growth Factor (VEGF) Therapies. Cureus.

[26] Weiss M., Sim D.A., Herold T., Schumann R.G., Liegl R., Kern C., Kreutzer T., Schiefelbein J., Rottmann M., Priglinger S. (2018). Compliance and adherence of patients with diabetic macular edema to intravitreal anti-vascular endothelial growth factor therapy in daily practice. Retina.

[27] Chen Y.P., Wu A.L., Chuang C.C., Chen S.N. (2019). Factors influencing clinical outcomes in patients with diabetic macular edema treated with intravitreal ranibizumab: Comparison between responder and non-responder cases. Sci. Rep..

[28] Usui-Ouchi A., Tamaki A., Sakanishi Y., Tamaki K., Mashimo K., Sakuma T., Ebihara N. (2021). Factors Affecting a Short-Term Response to Anti-VEGF Therapy in Diabetic Macular Edema. Life.

[29] Peto T., Akerele T., Sagkriotis A., Zappacosta S., Clemens A., Chakravarthy U. (2022). Treatment patterns and persistence rates with anti-vascular endothelial growth factor treatment for diabetic macular oedema in the UK: A real-world study. Diabet. Med..

[30] Holekamp N.M., Campbell J., Almony A., Ingraham H., Marks S., Chandwani H., Cole A.L., Kiss S. (2018). Vision Outcomes Following Anti–Vascular Endothelial Growth Factor Treatment of Diabetic Macular Edema in Clinical Practice. Am. J. Ophthalmol..

[31] Kiss S., Liu Y., Brown J., Holekamp N.M., Almony A., Campbell J., Kowalski J.W. (2014). Clinical utilization of anti-vascular endothelial growth-factor agents and patient monitoring in retinal vein occlusion and diabetic macular edema. Clin. Ophthalmol..

[32] Ciulla T.A., Pollack J.S., Williams D.F. (2021). Visual acuity outcomes and anti-VEGF therapy intensity in diabetic macular oedema: A real-world analysis of 28 658 patient eyes. Br. J. Ophthalmol..

[33] Virgili G., Curran K., Lucenteforte E., Peto T., Parravano M. (2023). Anti-vascular endothelial growth factor for diabetic macular oedema: A network meta-analysis. Cochrane Database Syst. Rev..

[34] Wells J.A., Glassman A.R., Ayala A.R., Jampol L.M., Bressler N.M., Bressler S.B., Brucker A.J., Ferris F.L., Hampton G.R., Jhaveri C. (2016). Aflibercept, Bevacizumab, or Ranibizumab for Diabetic Macular Edema: Two-year Results from a Comparative Effectiveness Randomized Clinical Trial. Ophthalmology.

[35] Wykoff C.C., Garweg J.G., Regillo C., Souied E., Wolf S., Dhoot D.S., Agostini H.T., Chang A., Laude A., Wachtlin J. (2024). KESTREL and KITE Phase 3 Studies: 100-Week Results With Brolucizumab in Patients With Diabetic Macular Edema. Am. J. Ophthalmol..

[36] Wykoff C.C., Abreu F., Adamis A.P., Basu K., Eichenbaum D.A., Haskova Z., Lin H., Loewenstein A., Mohan S., Pearce I.A. (2022). Efficacy, durability, and safety of intravitreal faricimab with extended dosing up to every 16 weeks in patients with diabetic macular oedema (YOSEMITE and RHINE): Two randomised, double-masked, phase 3 trials. Lancet.

