# Supplementary material for: Visual Acuity Outcomes and Influencing Factors in a Cohort of UK Real-World Diabetic Macular Oedema Patients During the First Two Years of Anti-VEGF Treatment
Source: Pharmaceutics. 2025 Jan 13;17(1):99. doi: 10.3390/pharmaceutics17010099 (PMC11769381; doi:10.3390/pharmaceutics17010099)
Supplement: Supplementary file 1 [file pharmaceutics-17-00099-s001.zip › pharmaceutics-3318110-supplementary.pdf]

Supplementary Material

Table S1: **Marginal Models with GEE for Analysing ISVA and Predictors including injection numbers in 1st and 2nd Year** (n=1035)

Characteristics	Univariate Analysis				Full model			
	1st year		2nd year		1st year		2nd year	
	**P-value**	**OR (95% CI)**	**P-value**	**OR (95% CI)**	**P-value**	**OR (95% CI)**	**P-value**	**OR (95% CI)**
**Age (years)**	0.202	0.99 (0.98, 1.00)	0.379	0.99 (0.98, 1.01)	0.042	0.98 (0.97, 0.99)	0.021	0.98 (0.97, 0.99)
**Gender-** Male	0.723	0.94 (0.70, 1.26)	0.286	1.19 (0.91, 1.56)	0.913	0.98 (0.73,1.32)	0.291	1.20 (0.91, 1.58)
**Diabetes Type**—Type II	0.771	0.935 (0.64,1.36)	0.263	1.26 (0.90, 1.76)	0.899	1.03 (0.69, 1.56)	0.175	1.36 (0.94, 1.98)
**Baseline Visual Acuity**:								
≥70 letters	(Ref)							
55-69 letters	0.003	1.89 (1.33,2.67)	0.035	1.50 (1.09, 2.05)	0.002	1.96 (1.36, 2.82)	0.017	1.59 (1.15,2.21)
35-54 letters	0.023	1.68 (1.15, 2.44)	0.035	1.56 (1.10, 2.20)	0.020	1.73 (1.18, 2.54)	0.017	1.68 (1.17,2.41)
<35 letters	0.50	1.25 (0.72, 2.17)	0.31	1.39 (0.82, 2.36)	0.377	1.36 (0.77, 2.41)	0.082	1.73 (0.99,3.00)
**Injection number:**								
≥7	(Ref)							
5-6	0.038	0.65 (0.47, 0.92)	0.015	0.43 (0.24, 0.76)	0.048	0.67 (0.48,0.93)	0.019	0.44 (0.25,0.78)
<5	0.013	0.58 (0.40, 0.83)	0.001	0.35 (0.21, 0.59)	0.032	0.62 (0.43,0.90)	0.001	0.35 (0.21,0.59)
**DR Grades:**								
Mild-moderate	(Ref)							
Moderate to Severe	0.746	1.08 (0.74,1.58)	0.92	0.95 (0.65, 1.40)	0.814	1.06 (0.70,1.61)	0.571	0.93 (0.61, 1.41)
PDR	0.746	0.93 (0.62,1.40)	0.068	0.66 (0.43, 1.0)	0.362	0.78 (0.50, 1.22)	0.011	0.57 (0.36, 0.91)

Table S2: Marginal models with GEE to analyse injection interval/number and other potential predictors associated with IM5LR in the 1st and 2nd year (n=1035) (Full model)

Characteristics	1st Year		2nd Year		Characteristics	1st Year		2nd Year	
	P-value	OR (95% CI)	P-value	OR (95% CI)		P-value	OR (95% CI)	P-value	OR (95% CI)
**Age (years)**	<0.001	0.98 (0.97, 0.99)	<0.001	0.98 (0.96,0.99)	**Age (years)**	<0.001	0.98 (0.97,0.99)	<0.001	0.97 (0.96, 0.98)
**Gender- Male**	0.743	0.95 (0.75, 1.21)	0.099	1.27 (1.00,1.61)	**Gender- Male**	0.938	1.01(0.80,1.28)	0.142	1.24 (0.97, 1.57)
**Diabetes Type—Type II**	0.040	1.46 (1.08, 1.99)	0.749	1.07 (0.77, 1.48)	**Diabetes Type—**Type II	0.028	1.51 (1.11, 2.05)	0.651	1.09 (0.79, 1.52)
**Baseline Visual Acuity**:					**Baseline Visual Acuity**:				
≥70 letters	(Ref)				≥70 letters	(Ref)			
55-69 letters	<0.001	2.96 (2.24, 3.90)	<0.001	2.45 (1.86, 3.21)	55-69 letters	<0.001	2.87 (2.18, 3.78)	<0.001	2.48 (1.88, 3.26)
35-54 letters	<0.001	3.56 (2.60, 4.89)	<0.001	3.26 (2.38, 4.48)	35-54 letters	<0.001	3.60 (2.61, 4.95)	<0.001	3.18 (2.32, 4.36)
<35 letters	<0.001	3.80 (2.34, 6.17)	<0.001	4.75 (2.79, 8.10)	<35 letters	<0.001	3.69 (2.26, 6.03)	<0.001	5.13 (3.03, 8.69)
**Injection Interval:**					**Injection number:**				
<8 weeks	(Ref)				≥7	(Ref)			
8 -11 weeks	0.014	0.64 (0.47, 0.86)	0.453	0.86 (0.62, 1.19)	5-6	0.004	0.63 (0.48,0.82)	0.537	0.85 (0.56, 1.30)
≥12 weeks	0.006	0.40 (0.23, 0.69)	<0.001	0.58 (0.45, 0.74)	<5	0.001	0.54 (0.40,0.72)	0.002	0.52 (0.36, 0.74)
**DR Grades:**					**DR Grades:**				
Mild-moderate	(Ref)				Mild-moderate	(Ref)			
Moderate to Severe	0.765	1.06 (0.79, 1.42)	0.564	1.11 (0.83, 1.47)	Moderate to Severe	0.909	1.02 (0.74,1.42)	0.759	1.06 (0.78, 1.44)
PDR	0.181	0.74 (0.51,1.07)	0.082	0.68 (0.47, 0.98)	PDR	0.171	0.72 (0.49,1.07)	0.042	0.62 (0.42, 0.91)

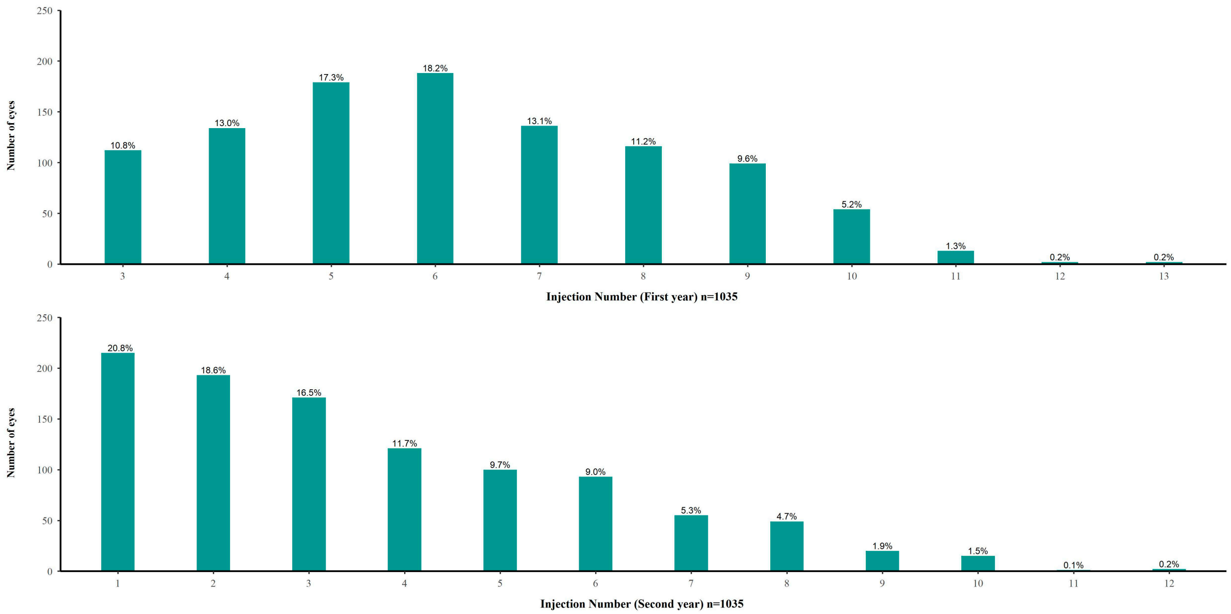

Figure S 1: The distribution of the number of injections in the first and second year
